# Alteration of EIF2 Signaling, Glycolysis, and Dopamine Secretion in Form-Deprived Myopia in Response to 1% Atropine Treatment: Evidence From Interactive iTRAQ-MS and SWATH-MS Proteomics Using a Guinea Pig Model

**DOI:** 10.3389/fphar.2022.814814

**Published:** 2022-01-28

**Authors:** Ying Zhu, Jing Fang Bian, Da Qian Lu, Chi Ho To, Carly Siu-Yin Lam, King Kit Li, Feng Juan Yu, Bo Teng Gong, Qiong Wang, Xiao Wen Ji, Hong Mei Zhang, Hong Nian, Thomas Chuen Lam, Rui Hua Wei

**Affiliations:** ^1^ Tianjin Key Laboratory of Retinal Functions and Diseases, Tianjin Branch of National Clinical Research Center for Ocular Disease, Eye Institute and School of Optometry, Tianjin Medical University Eye Hospital, Tianjin, China; ^2^ Centre for Myopia Research, School of Optometry, The Hong Kong Polytechnic University, Hong Kong SAR, China; ^3^ Centre for Eye and Vision Research (CEVR), Hong Kong SAR, China; ^4^ Research Centre for SHARP Vision (RCSV), The Hong Kong Polytechnic University, Hong Kong SAR, China; ^5^ Department of Ophthalmology, Tianjin Medical University General Hospital, Tianjin, China; ^6^ Shenzhen Research Institute, The Hong Kong Polytechnic University, Shenzhen, China

**Keywords:** Retina, iTRAQ-MS, SWATH-MS, FDM, Guinea pigs, Atropine

## Abstract

**Purpose:** Atropine, a non-selective muscarinic antagonist, effectively slows down myopia progression in human adolescents and several animal models. However, the underlying molecular mechanism is unclear. The current study investigated retinal protein changes of form-deprived myopic (FDM) guinea pigs in response to topical administration of 1% atropine gel (10 g/L).

**Methods:** At the first stage, the differentially expressed proteins were screened using fractionated isobaric tags for a relative and absolute quantification (iTRAQ) approach, coupled with nano-liquid chromatography-tandem mass spectrometry (nano-LC-MS/MS) (*n* = 24, 48 eyes) using a sample pooling technique. At the second stage, retinal tissues from another cohort with the same treatment (*n* = 12, 24 eyes) with significant ocular changes were subjected to label-free sequential window acquisition of all theoretical mass spectra (SWATH-MS) proteomics for orthogonal protein target confirmation. The localization of Alpha-synuclein was verified using immunohistochemistry and confocal imaging.

**Results:** A total of 1,695 proteins (8,875 peptides) were identified with 479 regulated proteins (FC ≥ 1.5 or ≤0.67) found from FDM eyes and atropine-treated eyes receiving 4-weeks drug treatment using iTRAQ-MS proteomics. Combining the iTRAQ-MS and SWATH-MS datasets, a total of 29 confident proteins at 1% FDR were consistently quantified and matched, comprising 12 up-regulated and 17 down-regulated proteins which differed between FDM eyes and atropine treated eyes (iTRAQ: FC ≥ 1.5 or ≤0.67, SWATH: FC ≥ 1.4 or ≤0.71, *p*-value of ≤0.05). Bioinformatics analysis using IPA and STRING databases of these commonly regulated proteins revealed the involvement of the three commonly significant pathways: EIF2 signaling; glycolysis; and dopamine secretion. Additionally, the most significantly regulated proteins were closely connected to Alpha-synuclein (SNCA). Using immunostaining (*n* = 3), SNCA was further confirmed in the inner margin of the inner nuclear layer (INL) and spread throughout the inner plexiform layer (IPL) of the retina of guinea pigs.

**Conclusion:** The molecular evidence using next-generation proteomics (NGP) revealed that retinal EIF2 signaling, glycolysis, and dopamine secretion through SNCA are implicated in atropine treatment of myopia in the FDM-induced guinea pig model.

## Introduction

Myopia (near-sightedness) has emerged as a worldwide public health issue (Morgan et al., 2012). High myopia (≤ 6.00 diopters) is associated with an increased risk of cataracts, glaucoma, and retinal detachment (Wong et al., 2014; Verkicharla et al., 2015; Saw et al., 2019). These high myopia-associated ocular complications may cause blindness or permanent visual loss. Although the exact molecular mechanism underlying myopia remains unclear, there are several routes of intervention for the prevention or delay of myopia progression, including the application of low dosage (0.01,0.5, and 1%) atropine (Chua et al., 2006; Chia et al., 2016; Yam et al., 2019), orthokeratology (Cho and Cheung, 2012; Hiraoka et al., 2018), and defocus lenses (Tse et al., 2005; Tse et al., 2007; Tse and To, 2011; McFadden et al., 2014; Lam et al., 2019).

Atropine is a non-selective muscarinic antagonist, which can assist in diagnosing refractive errors in young children (Wildsoet et al., 2019). The inhibitory effect of atropine on myopia progression has been found in different animal models, including the tree shrew (Wan et al., 2018), monkey (Raviola and Wiesel, 1985; Kiorpes et al., 1987; Tigges et al., 1999), and chick (Gallego et al., 2012), as well as in young children (Chia et al., 2012; Huang et al., 2016; Yam et al., 2019). However, its underlying molecular mechanism is still unclear. Animal studies have supported the theory that biochemical signal cascades are initialized from the retina in response to defocused visual stimuli to regulate eye growth through tissue remodeling at the sclera (Morgan, 2003; Wallman and Winawer, 2004; Troilo et al., 2019). Retinal neurotransmitters have been suggested to regulate eye growth based on animal studies. The retinal dopamine (DA) level was decreased in form-deprived myopia (FDM) animal models, including in chicks (Stone et al., 1989), rhesus monkeys (Iuvone et al., 1989), guinea pigs (Mao et al., 2011), and tree shrews (McBrien et al., 2001). A reduced retinal DA level was also found in Lens-induced myopia (LIM) chicks (Guo et al., 1995). Critical inhibitory neurotransmitters, antagonists of γ-aminobutyric acid (GABA) receptor, inhibited eye growth in the FDM model of chicks (Stone et al., 2003), while increased expression of GABA was found in the retinas of LIM guinea pigs (Zhao et al., 2017). In contrast, the release of retinal DA was increased in FDM chicks after atropine treatment (Schwahn et al., 2000), and the retinal levels of GABA transporter 1 were also significantly decreased after atropine treatment (Barathi et al., 2014).

Proteomic approaches have become powerful tools to screen thousands of protein candidates simultaneously, which enables the detection of global regulation of protein expression (Pandey and Mann, 2000). Data-dependent acquisition (DDA) is a popular strategy in shotgun proteomics for target screening. Isobaric tags for relative and absolute quantification (iTRAQ) has remained a well-established multiplexing DDA approach for biomarker discovery in quantitative proteomics. However, limitations of DDA include suboptimal quantification and reproducibility (Liu et al., 2004; Michalski et al., 2011), under-sampling, and a bias towards high abundance proteins or peptides (Kiyonami et al., 2011). To overcome the limitations in DDA, data-independent acquisition (DIA) has rapidly gained attention as an alternative label-free strategy. In DIA, data reproducibility between technical replicates is increased, and low abundance precursors are better represented (Geromanos et al., 2009). DIA is successfully applied in the TripleTOF system (SCIEX), also termed SWATH (Gillet et al., 2012). The SWATH-MS based proteomics approach has become an increasingly popular proteomics platform (Gillet et al., 2012) for both clinical and basic research studies (Anjo et al., 2017), with broad applications in biomarker discovery and understanding of biological mechanisms (Simpson et al., 2009; Brown, 2014; Selevsek et al., 2015; Ortea et al., 2016; Anjo et al., 2017). It was also used to study retinal protein regulation during normal ocular growth (Shan et al., 2018a) and LIM (Bian et al., 2021) guinea pigs by our group. Moreover, it can be applied for the orthogonal verification of targets screened from the genomic data in cancer disease (Zhang et al., 2016).

To date, only one study has attempted the investigation of retinal protein changes after atropine treatment in LIM mouse model using an iTRAQ-MS based proteomics approach (Barathi et al., 2014). Two other mRNA expression studies investigated atropine-treated human scleral fibroblasts (Hsiao et al., 2019) and corneal epithelial cells (Chang et al., 2019). Hence, the knowledge of the retinal protein changes after atropine treated myopia was very minimal. The main objective of the present work was to characterize the whole retinal proteome and investigate differentially expressed proteins, which could reveal the key molecular changes in FDM guinea pigs in response to atropine treatment. It also investigated the feasibility of using combined iTRAQ-MS and SWATH-MS protocols for other retinal diseases.

## Results

### Refractive Errors and Ocular Dimensions Changes

**FIGURE 1:**
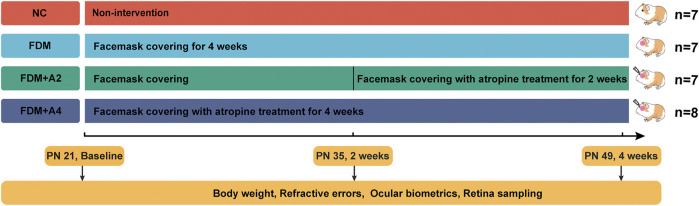
The time points selected for biometric measurements, and the total number of animals included at each time point for iTRAQ-MS based proteomics study.

**FIGURE 2:**
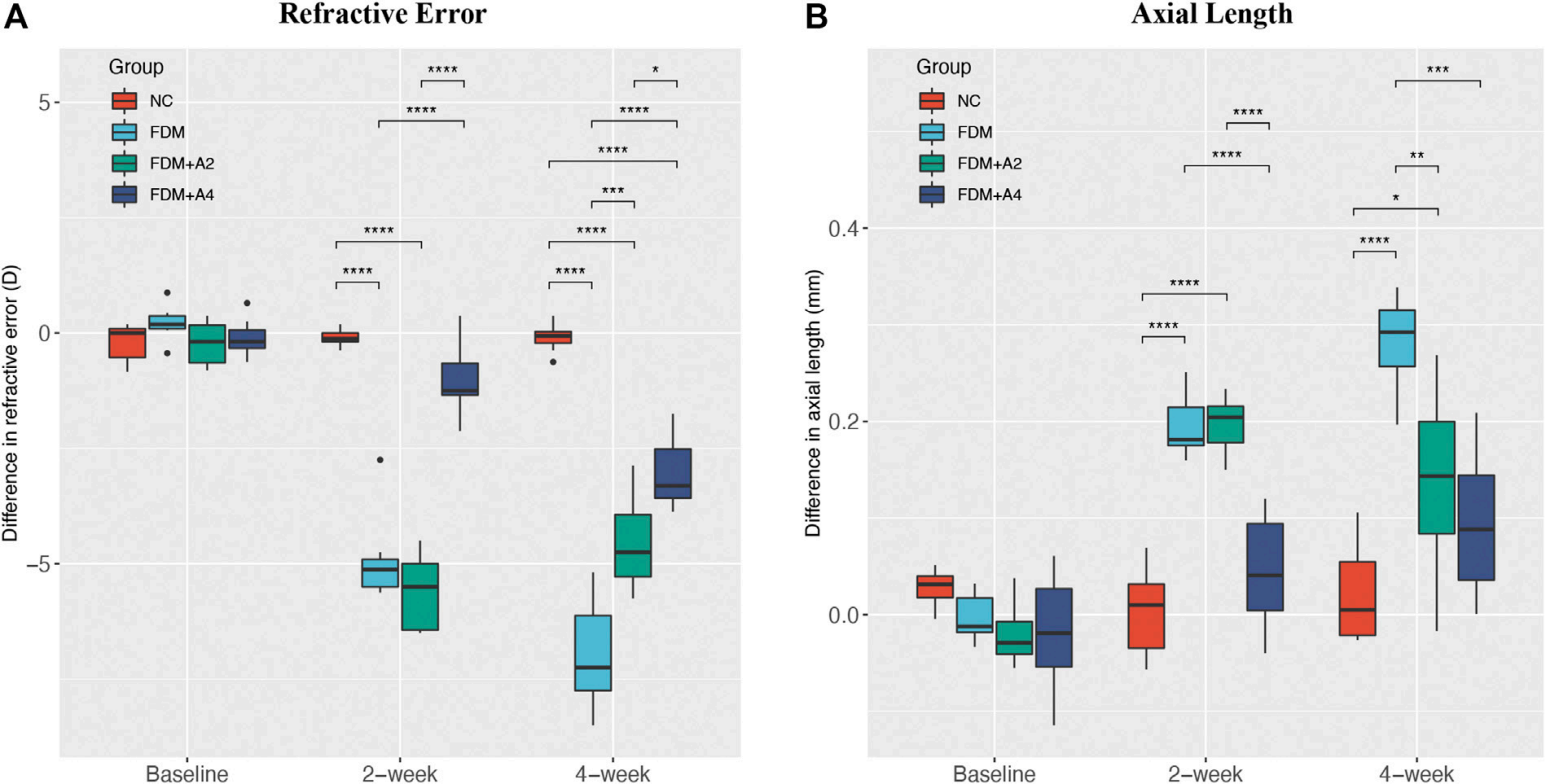
The effect of different treatments on the mean difference between the lens-wearing and fellow eye in **(A)** refractive error, **(B)** axial length (AL) at baseline, after 2-weeks (FDM + A2) and 4-weeks (FDM + A4) atropine treatment compared with normal control (NC) (*n* = 7), FDM (*n* = 7), FDM + A2 (*n* = 7), FDM + A4 (*n* = 8) and groups. (mean ± SD, Two-way mixed-design ANOVA with Bonferroni multiple comparison, *p < 0.05, **p < 0.01, ***p < 0.001).

During the 4-weeks study period, guinea pigs were randomly divided into four individual groups: the normal control group (NC, *n* = 7), the monocularly form-deprived myopia group (FDM, *n* = 7), the FDM with 2-weeks atropine-treatment group (FDM + A2, *n* = 7), and the FDM with 4-weeks atropine-treatment group (FDM + A4, n = 8) (Figure 1). In general, there were no significant differences between the two eyes at baseline with respect to refractive error and other ocular parameters among the four groups. After 2-weeks FDM treatment, relative myopic changes were found in the FDM group (-4.902 ± 0.997 D, mean ± SD), FDM + A2 group (-5.625 ± 0.845 D, mean ± SD), and FDM + A4 group (−1.031 ± 0.773 D, mean ± SD) compared to control eyes, as expected. The mean refractive error changes (treated eyes minus control eyes) also showed significant differences among the normal control, FDM, FDM + A2, and FDM + A4 groups (two-way mixed-design ANOVA, all of *p* < 0.001, Supplementary Table S1, Figure 2A). In addition, less myopia was found in the FDM + A4 group compared to the FDM group (FDM + A4 *vs* FDM: −1.031 ± 0.773 D *vs.* −4.902 ± 0.997 D, mean ± SD, *p* = 3.03e-09, Figure 2A). Compared to contralateral control eyes, A-scan also confirmed the corresponding elongation of the vitreous chamber depth (VCD) (*p* < 0.001) and axial length (AL) in the treated eyes (*p* < 0.001, Figure 2B) in the FDM, FDM + A2, and FDM + A4 groups. Notably, the means of VCD (FDM + A4 *vs* FDM: 0.014 ± 0.017 mm *vs* 0.081 ± 0.017 mm, mean ± SD, *p* = 1.37e-06) and AL difference (FDM + A4 *vs* FDM: 0.044 ± 0.056 mm *vs* 0.196 ± 0.033 mm, mean ± SD, *p* = 2.77e-06, Figure 2B) in the FDM + A4 group were significantly smaller than the changes in the FDM group, which was consistent with the finding for refractive error.

The 4-weeks treatment also resulted in similar changes of ocular parameters towards myopia in FDM, FDM + A2, and FDM + A4 groups to the 2-weeks treatment. Significantly less myopia was found in the FDM + A4 group compared to the FDM group (FDM + A4 *vs* FDM: −3.047 ± 0.737 D *vs.* −6.955 ± 1.194 D, mean ± SD, *p* < 0.001). Correspondingly, significantly smaller changes of axial length (FDM + A4 *vs* FDM: 0.094 ± 0.075 mm *vs* 0.282 ± 0.049 mm, *p* < 0.001, Figure 2B) were also observed in the FDM + A4 group compared to the FDM group. In addition, compared to the FDM + A2 group, a smaller elongation of AL (−0.043 mm) and enlargement of the VCD (−0.009 mm) were observed in the FDM + A4 group, although the differences did not reach statistical significance (*p* > 0.05, Figure 2B). However, less myopia was observed in the FDM + A4 group, compared with the FDM + A2 group (FDM + A4 *vs* FDM + A2: -3.047 ± 0.737 D *vs.* -4.545 ± 1.053 D, mean ± SD, *p* = 0.019, Figure 2A).

### Protein Identification and Gene Ontology Function Classification Analysis Using the iTRAQ-MS Approach

**FIGURE 3:**
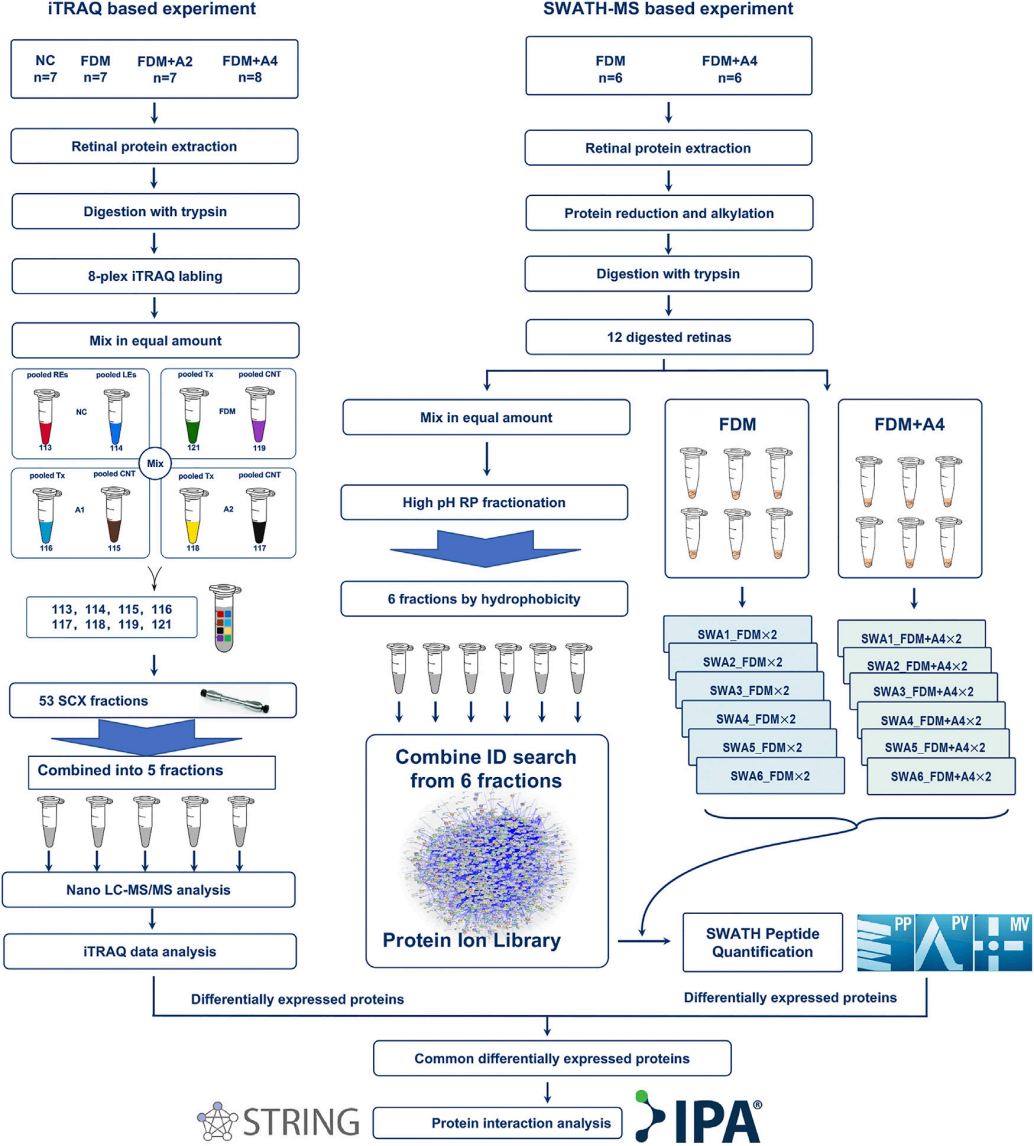
Schematic workflow of quantitative proteomics using iTRAQ-MS and SWTAH-MS proteomics to study effects on protein regulation of atropine treatment on myopic (FDM) eyes. Overall, six individual retinal samples from each time point were equally pooled to form representative retinal lysates of specific groups using an iTRAQ based proteomic approach. Another new batch of six individual retinas was selected in the validated experiment using a SWATH based proteomic approach. The 2 μg digested protein from each sample was used as two technical replicates under SWATH-MS. Protein identification and quantification were performed using ProteinPilot, PeakView, and MarkerView software, followed by online bioinformatics analysis.

**FIGURE 4:**
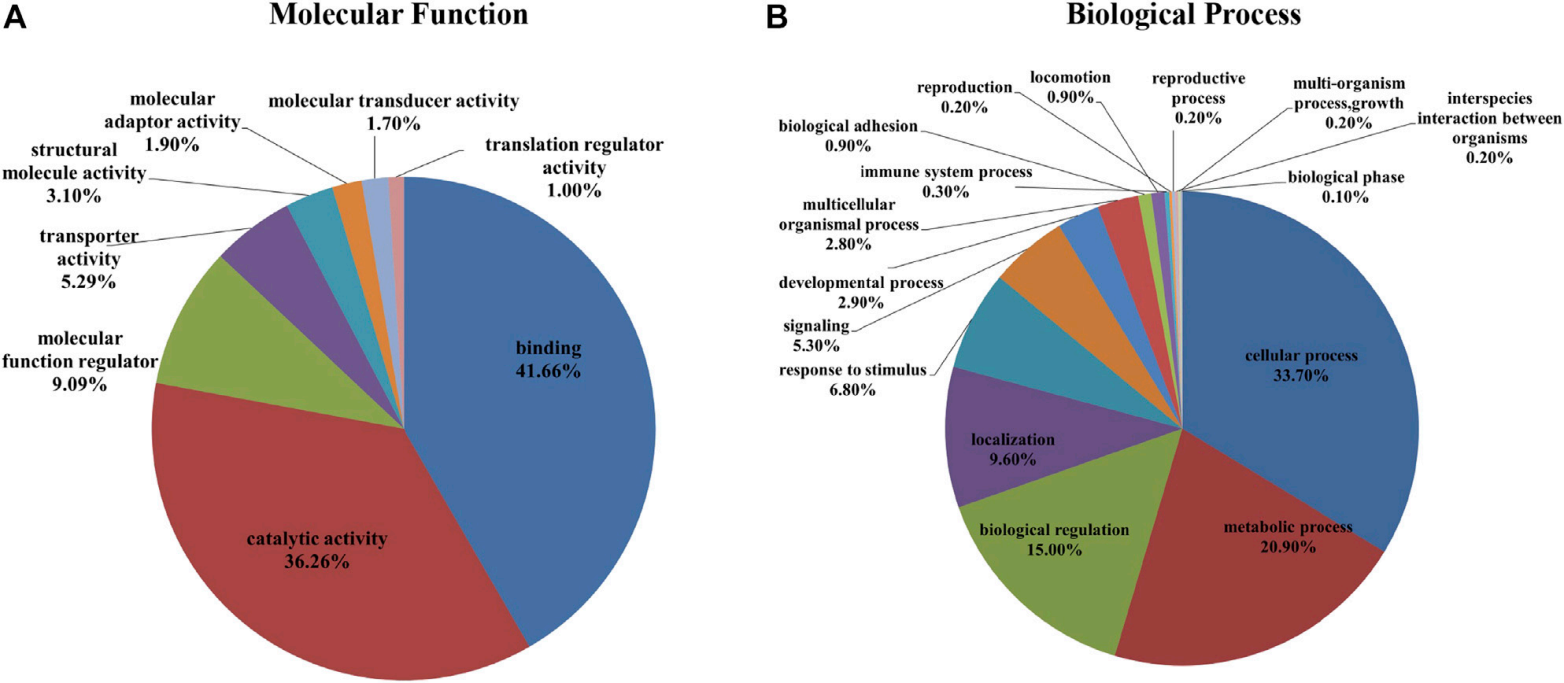
A total of 1,695 identified proteins (1,545 gene names) were annotated using the PANTHER™ Classification System according to their molecular function **(A)** and biological process **(B)**.

In the iTRAQ-MS approach, retinal lysates of age-matched animals from each group (NC, FDM, FDM + A2, and FDM + A4) were labelled with different iTRAQ reagents and pooled together for MS analysis (Left panel in Figure 3). The protein identification using this approach, resulted in the identification of 1,695 proteins (8,875 peptides) at 1% global false discovery rate (FDR) by the DDA approach. Of these, 1,545 proteins (∼90%) could be successfully converted to relevant guinea pigs’ gene names using the Uniprot database (https://www.uniprot.org/) followed by Gene Ontology (GO) classification and analysis using the PANTHER™ database (http://www.pantherdb.org/). Among all the mapped gene IDs, the top three molecular functions of retinal proteins were “binding” (GO: 0005488) (43.70%), “catalytic activity” (GO: 0003824) (37.50%), and “molecular function regulator” (GO: 0098772) (5.80%) (Figure 4A), whilst for biological processes, “cellular process” (GO: 0009987) (35.40%), “metabolic process” (GO: 0008152) (21.70%), and “biological regulation” (GO: 0065007) (14.10%) were identified as the major three groups in the retinal proteome (Figure 4B).

### Protein Quantitation Using the iTRAQ-MS Approach

For quantitative analysis, a cut-off ratio of ≥1.5 or ≤0.67-fold change was considered differential expression. Two main comparisons were performed to explore the temporal atropine effect on FDM eyes, including the FDM + A2 group (FDM eyes with 2 weeks atropine treatment) *vs* FDM group (FDM eyes) and FDM + A4 group (FDM eyes with 4 weeks atropine treatment) *vs* FDM group (FDM eyes), respectively. Four-hundred and sixty-six differentially expressed proteins were initially found in the FDM + A2 group compared to the FDM group. To avoid the potential confounding factor of intrinsic protein difference (intra-ocular difference) between the right and left eyes, the same differentially expressed proteins, found between the two eyes in the normal control group (NC) were excluded. Also, as some key molecular signals are expected to be expressed in opposite directions between the myopic and atropine treated eyes, as reported by an earlier study using the LIM mouse (Barathi et al., 2014), several proteins with the same expression direction in FDM + A2 *vs* FDM group and FDM *vs* FDM_C group (contralateral control eyes in FDM group) were excluded, allowing the targeted protein list to be more specific to atropine treatments. Finally, 310 differentially expressed proteins (306 gene names) were considered as positive findings in the FDM + A2 *vs* FDM group (Supplementary Table S2). Similarly, 668 differentially expressed proteins were initially found following comparison of the FDM + A4 group to the FDM group, using the same cut-off ratio. After applying the same filtering criteria as in the FDM + A2 group, 479 differentially expressed proteins (469 gene names) were considered as more specifically regulated proteins in the FDM + A4 *vs* FDM group (Supplementary Table S3).

### Protein Identification and Gene Ontology Function Classification Analysis Using Sequential Window Acquisition of all Theoretical Mass Spectra Approach for Confirmation

To further confirm only highly confident candidates altered in response to atropine treated in FDM guinea pig, another new batch of retinas in the FDM group (n = 6) and FDM + A4 group (n = 6) were collected for further validation using SWATH-MS analysis (Right panel in Figure 3). Six animals (12 retinas) from the FDM + A4 and FDM groups were included in the statistical analysis with individual MS injection. Similar ocular biometrics were found in this new batch of animals (Supplementary Table S4) as in the iTRAQ experiment. To build a comprehensive ion library for extracting SWATH-MS files, pooled peptides from all 12 guinea pigs (FDM + A4 group and FDM group, *n* = 6 per group) were divided into six fractions using the High pH Reversed-Phase peptide fractionation technique. After combining the search of six separate IDA injections, a total of 5,961 proteins (51,871 peptides) were identified in this comprehensive ion library. All the raw MS files in iTRAQ-MS and SWATH-MS were subsequently published in Data in Brief (Zhu et al., 2020) and saved in the PeptideAtlas public repository (Desiere et al., 2006) with the accession number of PASS01507 for open access (http://www.peptideatlas.org/).

**FIGURE 5:**
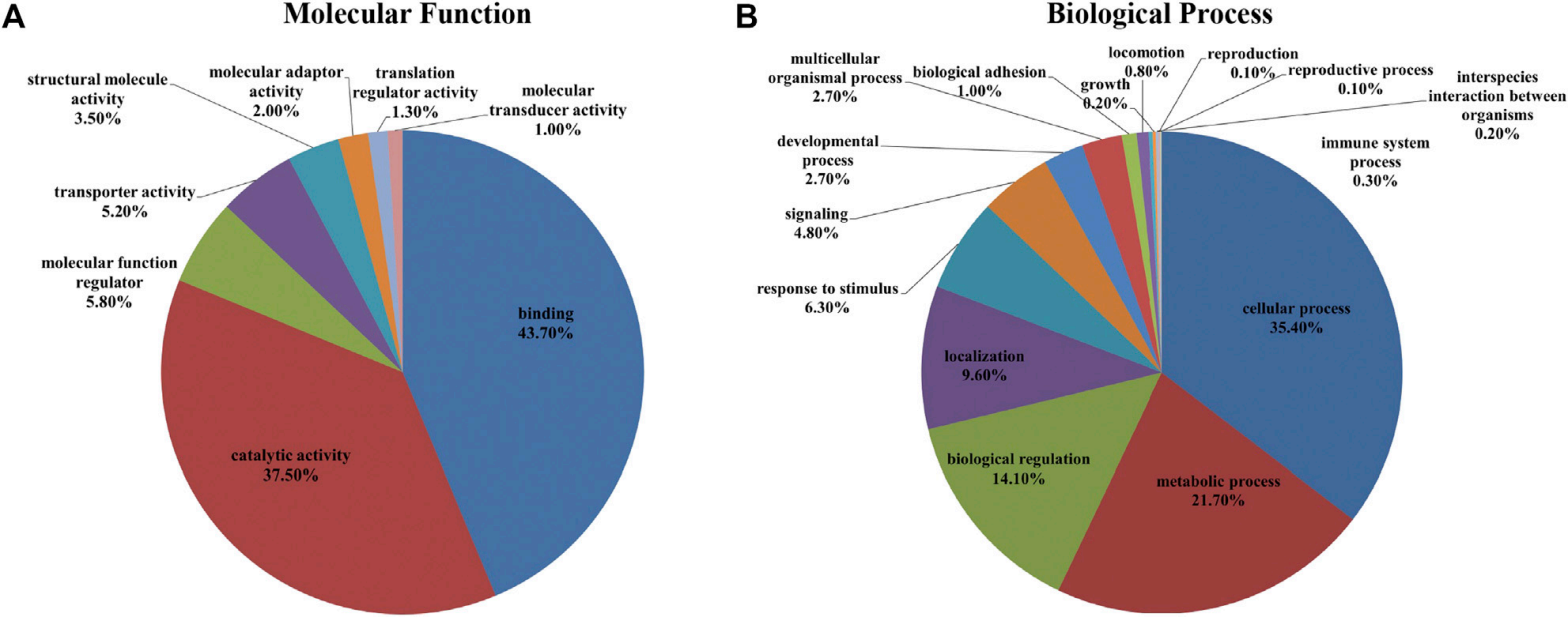
A total of 5,961 identified proteins (5,830 gene names) were annotated with the PANTHER™ Classification System (www.pantherdb.org) according to their molecular function **(A)** and biological process **(B)**.

The identified proteins were loaded in the Uniprot database, which resulted in 5,830 proteins being converted to guinea pig gene names. To better understand the fundamental protein functions of the comprehensive retinal proteome, GO function classification was performed again using the PANTHER ™ classification system. According to the GO analysis, the top three molecular functions of retinal proteins were binding (GO: 0005488) (41.66%), catalytic activity (GO: 0003824) (36.26%), and molecular function regulator (GO: 0098772) (9.09%) (Figure 5A), while the major biological processes included cellular process (GO: 0009987) (33.70%), metabolic process (GO: 0008152) (20.90%), and biological regulation (GO: 0065007) (15.00%) (Figure 5B).

### Protein Quantitation Using Sequential Window Acquisition of all Theoretical Mass Spectra Approach

**FIGURE 6:**
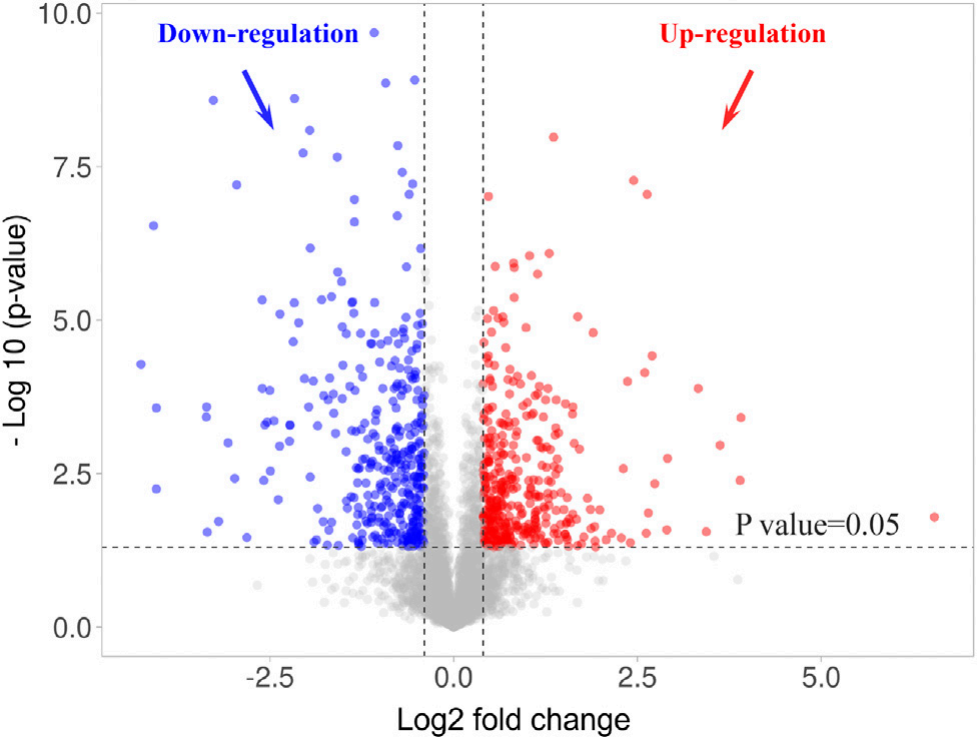
Volcano plot of 774 differentially expressed retinal proteins in 4-weeks atropine treated eyes (FDM + A4 group) compared to 4-weeks FDM eyes (FDM group) using a SWATH-MS based proteomics approach. Criteria for significant differential expression were as log2 ratio (FDM + A4/FDM) ≥ 0.43 or ≤ -0.43 (∼1.40FC), *p*-value ≤ 0.05, welch T-test.

Considering the high repeatability of SWATH-MS based proteomics with a lower coefficient of variation (CV) in quantification (Collins et al., 2017) and a similar guinea pig retinal proteome study using SWATH-MS (Shan et al., 2018a), a slightly lower cut-off value was selected for this study (FDM + A4 *vs* FDM: ≥1.4 or ≤0.71 *p*-value of ≤0.05, welch T-test). After quantitative analysis, 774 proteins (714 gene names) were significantly changed when atropine treated eyes were compared to FDM eyes (Supplementary Table S5). VolcaNoseR (Goedhart and Luijsterburg, 2020) was used to generate a volcano plot, which showed the differentially expressed proteins (Figure 6). The fold change (FDM + A4 *vs* FDM) was converted to a log2 fold change in the x-axis, and the *p*-value was converted to log10 *p*-value in the y-axis. The details of differentially expressed proteins were shown in Supplementary Table S5. To better understand these significantly regulated proteins, we performed GO function classification using the PANTHER system again. Among all the mapped gene ID, the top three molecular functions of retinal proteins were catalytic activity (GO: 0003824) (35.40%), binding (GO: 0005488) (41.70%), and molecular function regulator (GO:0098772) (9.20%) whereas cellular process (GO: 0009987) (34.70%), metabolic process (GO: 0008152) (22.00%), and biological regulation (GO:0065007) (14.10%) were the top three biological processes (Supplementary Figure S1).

### Comparison of Commonly Regulated Proteins in the FDM + A4 and FDM Groups Using Both Approaches

Changes in protein expression in response to atropine treatment (FDM + A4 group *vs* FDM group), were observed in 50 proteins found by both the iTRAQ-MS and SWATH-MS approaches. Of these, proteins with expression regulation in the same direction by both methods were of particular interest, as they may be considered to represent the retinal response to atropine treatment during myopia progression with high confidence. As a result, 29 similarly differentially expressed proteins (12 up-regulation and 17 down-regulation) were considered highly confident targets for further analysis (Table 1).

**TABLE 1:** Twenty-nine commonly regulated proteins with the same directional expression change found in the FDM + A4 group compared to the FDM group by both the iTRAQ-MS and SWATH-MS approach meeting criteria for differential expression (iTRAQ: FC ≥ 1.5 or ≤0.67, SWATH: n = 6, FC ≥ 1.4 or ≤0.71, *p*-value of ≤0.05, welch T-test). Red represents up-regulated proteins and blue down-regulated proteins.

NO.	Uniprot entry names	Gene name	Protein name	iTRAQ-MS		SWATH-MS	
				FDM + A2/FDM	FDM + A4/FDM	FDM + A4/FDM	*p* value
1	A0A286Y0L7	PKM	Pyruvate kinase	0.75	0.30	0.46	0.002
2	H0VPZ2_CAVPO	SNCA	Alpha-synuclein	0.95	0.39	0.21	0.001
3	H0UTZ2_CAVPO	MACROH2A1	Core histone macro-H2A	1.00	0.42	0.62	<0.001
4	H0WBS4_CAVPO	NUCKS1	Nuclear casein kinase and cyclin dependent kinase substrate 1	0.85	0.45	0.05	<0.001
5	H0W6L0_CAVPO	CDS2	Phosphatidate cytidylyltransferase	0.81	0.48	0.68	<0.001
6	H0W636_CAVPO	RPL13	60S ribosomal protein L13	1.67	0.49	0.53	0.021
7	H0UTH3_CAVPO	NECAP1	NECAP endocytosis associated 1	0.75	0.50	0.70	<0.001
8	A0A286XMP4_CAVPO	HNRNPD	Heterogeneous nuclear ribonucleoprotein D	0.67	0.55	0.51	0.011
9	A0A286XH94_CAVPO	GTF2I	General transcription factor IIi	0.82	0.56	0.18	<0.001
10	H0W051_CAVPO	SNCG	Gamma-synuclein	0.70	0.56	0.49	0.002
11	H0W577_CAVPO	BSG	Basigin	1.79	0.58	0.14	0.035
12	H0VRE0_CAVPO	SEC14L2	SEC14 like lipid binding 2	0.69	0.61	0.63	0.004
13	A0A286Y4P1_CAVPO	SEL1L	SEL1L adaptor subunit of ERAD E3 ubiquitin ligase	0.82	0.62	0.39	0.035
14	A0A286XH98_CAVPO	LOC100717315	Glutathione transferase	1.08	0.65	0.66	0.006
15	A0A286XWY9_CAVPO	XPNPEP1	X-prolyl aminopeptidase 1	1.13	0.66	0.71	0.002
16	A0A286XY99_CAVPO	LOC100734633	MFS domain-containing protein	0.83	0.67	0.71	<0.001
17	H0UZK2_CAVPO	SNAP25	Synaptosomal-associated protein	1.18	0.67	0.23	<0.001
18	H0UYD8_CAVPO	AP3M2	AP-3 complex subunit mu-2	0.88	1.54	1.41	0.004
19	H0UWL2_CAVPO	GSTZ1	Glutathione S-transferase zeta 1	8.24	1.58	1.55	<0.001
20	A0A286X9V5_CAVPO	ABHD11	Abhydrolase domain containing 11	0.79	2.00	1.46	0.050
21	H0W551_CAVPO	RPS19	40S ribosomal protein S19	1.06	2.01	1.39	0.002
22	A0A286XAQ3_CAVPO	TOMM22	Mitochondrial import receptor subunit TOM22 homolog	1.74	2.09	1.37	0.026
23	A0A286XTA4_CAVPO	MYEF2	Myelin expression factor 2	2.05	2.25	1.38	<0.001
24	H0VZ48_CAVPO	SERBP1	SERPINE1 mRNA binding protein 1	0.55	2.49	1.37	0.001
25	H0V3E4_CAVPO	POLR2C	RNA polymerase II subunit C	1.66	2.70	12.34	0.001
26	H0V5J3_CAVPO	PIP4K2B	PIPK domain-containing protein	3.13	3.37	1.39	<0.001
27	A0A286Y4G4_CAVPO	KRT3	Keratin 3	1.33	4.97	1.95	0.030
28	H0VFF0_CAVPO	RPS7	40S ribosomal protein S7	1.80	5.15	1.67	<0.001
29	A0A286Y0B2_CAVPO	RAB10	RAB10, member RAS oncogene family	5.97	5.81	1.56	0.001

**T:** treatment eyes; **C:** control eyes; **FDM:** form-deprived myopia group; **FDM + A2:** form-deprived myopia with 2-weeks atropine treatment group; **FDM + A4:** form-deprived myopia with 4-weeks atropine-treatment group.

### Pathways and Protein Interaction Analysis of 29 Commonly Regulated Proteins

To identify pathways for biological insights into control of ocular growth mechanisms using atropine, the common 29 proteins were uploaded to The Ingenuity Pathway Analysis (IPA) for pathway analysis. The *p*-value was calculated with Fischer’s exact test, which reflected the likelihood that the association between a set of genes in our dataset and the canonical pathway was significant. Significant pathways were defined as *p*-values of less than 0.05. A total of 16 significant pathways were predicted by pathways analysis (Table 2). Among the significant pathways, the most significant was “EIF2 Signaling”.

**TABLE 2:** Sixteen significant pathways with associated proteins were predicted by IPA from 29 differentially expressed proteins using iTRAQ and SWATH analysis. Criteria for significant pathways were as -lg (*p*-value) ≥ 1.3 (*p*-value <0.05), Fischer’s exact test.

No	Ingenuity Canonical Pathways	-lg (*p*-value)	Associated gene(s) identified
1	EIF2 Signaling	2.66	RPL13, RPS19, RPS7
2	Huntington’s Disease Signaling	2.38	POLR2C, SNAP25, SNCA
3	Synaptogenesis Signaling Pathway	2.25	SNAP25, SNCA, SNCG
4	Tyrosine Degradation I	2.24	GSTZ1
5	Sumoylation Pathway	2.19	SERBP1, SNCA
6	Regulation of eIF4 and p70S6K Signaling	1.74	RPS19, RPS7
7	Parkinson’s Signaling	1.73	SNCA
8	Coronavirus Pathogenesis Pathway	1.63	RPS19, RPS7
9	mTOR Signaling	1.60	RPS19, RPS7
10	CDP-diacylglycerol Biosynthesis I	1.60	CDS2
11	Phosphatidylglycerol Biosynthesis II	1.56	CDS2
12	Glutathione Redox Reactions I	1.54	GSTZ1
13	D-myo-inositol (1,4,5)-Trisphosphate Biosynthesis	1.52	PIP4K2B
14	Glycolysis I	1.52	PKM
15	Glutathione-mediated Detoxification	1.42	GSTZ1
16	Nucleotide Excision Repair Pathway	1.40	POLR2C

**FIGURE 7:**
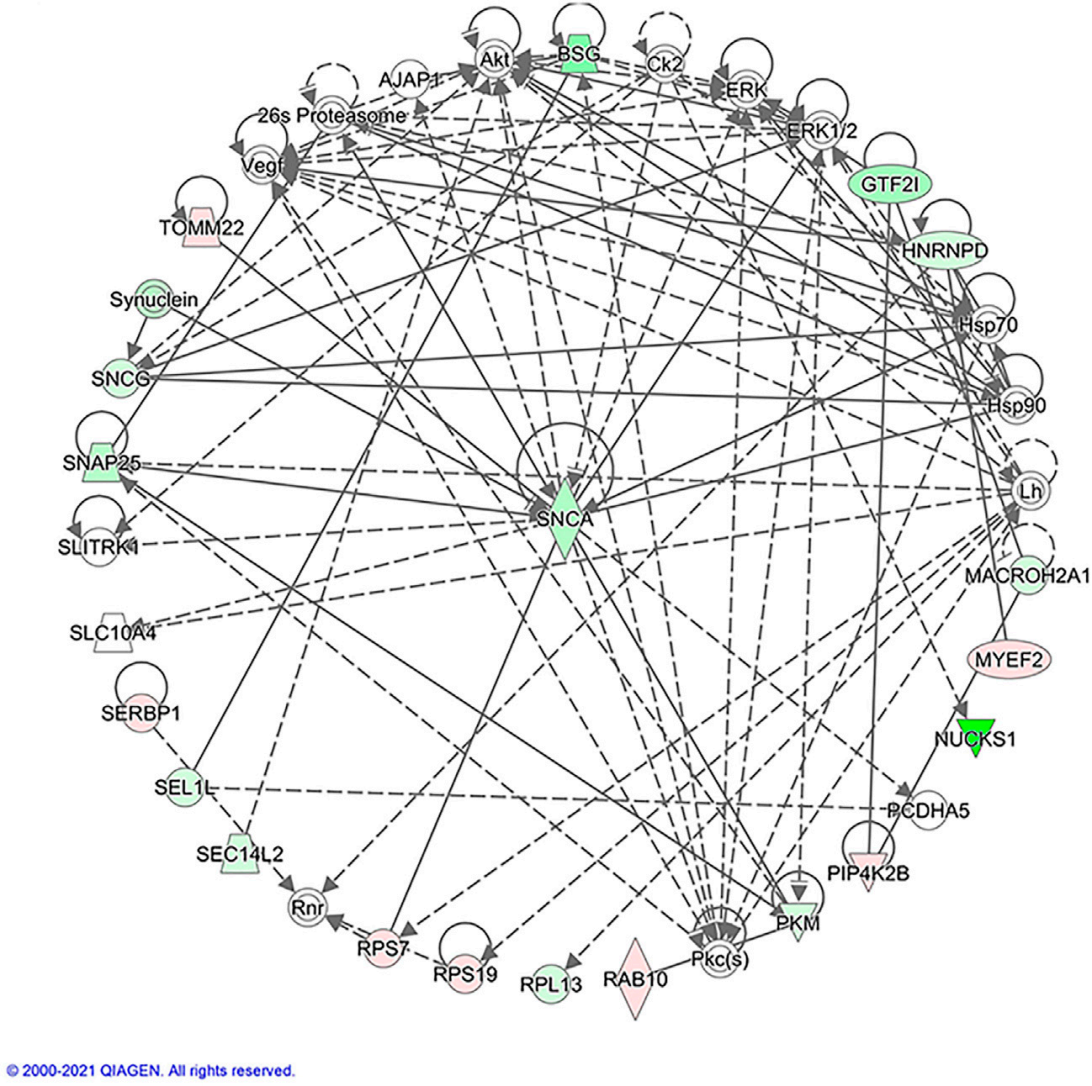
Protein-protein interaction analysis using IPA. Red represents up-regulated genes, and green down-regulated genes. The un-colored genes indicate additional linked proteins of this network that were not detected by the proteomics analysis. The solid lines represent a robust correlation with partner proteins, and dashed lines represent statistically significant, but less frequent correlations. The protein-protein interactions are indicated by arrows. The different shape nodes indicate different protein functions: enzymes (diamond); nuclear receptors (rectangle); transcription regulators (oval); cytokines (square); transporter (trapezoid); and others (circle).

IPA was further used to analyze the protein-protein interactions associated with atropine treatment. The regulated proteins were highlighted in different colors (Figure 7). In addition, the most significant protein-protein interactions are also shown. These proteins formed a circle cluster, in which the majority of proteins were connected to Alpha-synuclein (SNCA), suggesting that SNCA may be the core candidate having a close interaction with other regulated proteins.

**FIGURE 8:**
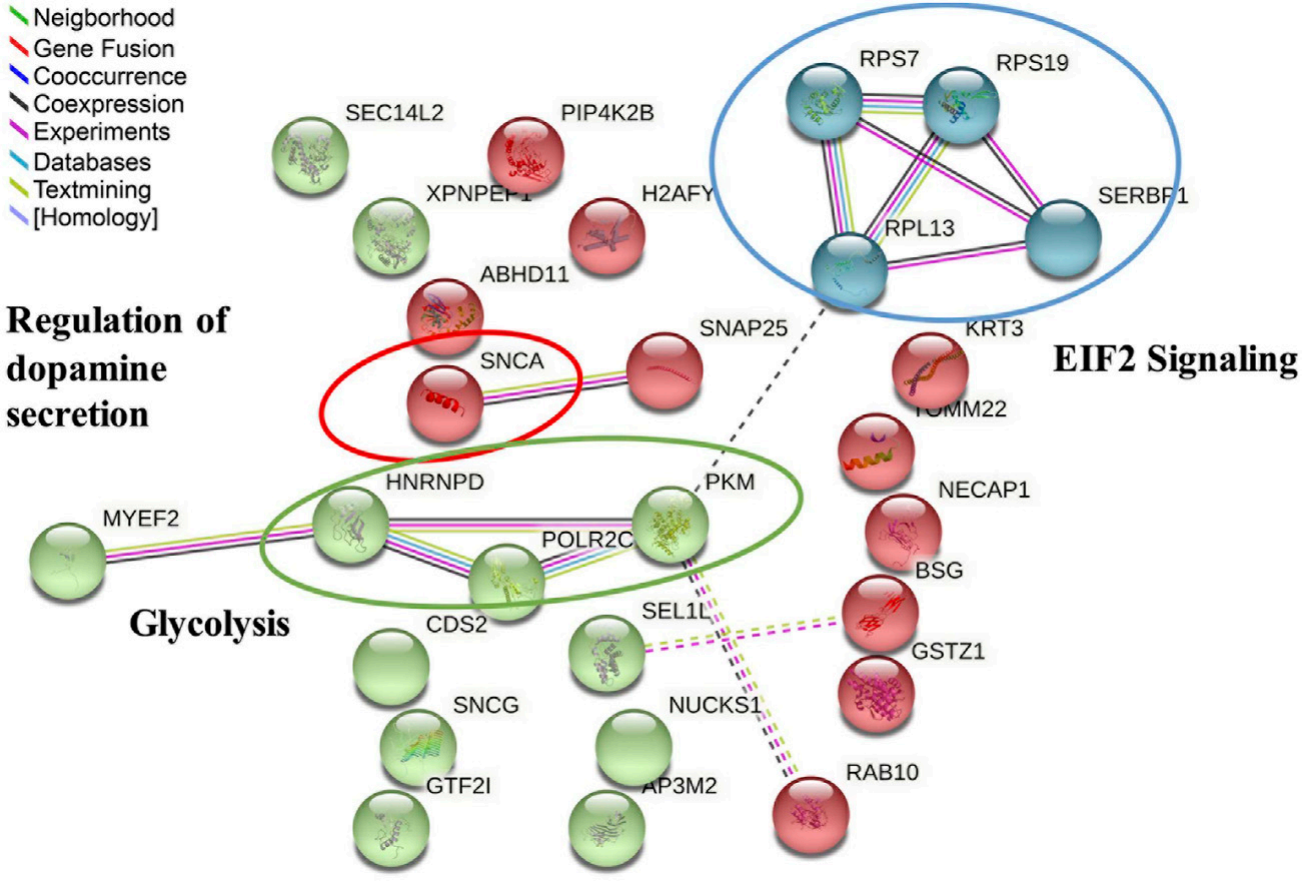
Protein–protein interaction analysis of 29 significantly regulated proteins in response to atropine treatment found by the STRING database after kmeans clustering, including 27 matched nodes (gene names) and 14 edges (predicted functional associations). Key molecular functions are circled and named in the network analysis. The three main interaction clusters are colored in red, green, and blue after use of the K-Means clustering algorithm.

In addition, the same 29 commonly regulated proteins were also uploaded to the STRING online database to study protein and protein interactions. A total of 27 nodes (gene names) and 14 edges (predicted functional associations) were observed (Figure 8). Among enriched biological processes, the top three processes were regulation of acyl-CoA biosynthetic process (GO:0050812) (0.67% FDR), neurotransmitter uptake (GO:0001504) (1.49%FDR), and regulation of dopamine secretion (GO:0014059) (2.74%FDR). Of these, SNCA was found to be associated with all the top three processes. After the application of the kmeans clustering algorithm, the results showed three main interaction clusters, including EIF2 signaling, regulation of dopamine secretion, and glycolysis clusters (Figure 8). Combining IPA and STRING analysis, SNCA was the most critical regulated protein, involved in multiple pathways and biological processes. The comparison between all the pathways enriched by IPA and interactions predicted by STRING also showed that EIF2 signaling, and glycolysis were the common significant pathways.

### Alpha-Synuclein Immunoreactivity Pattern in the Retina

**FIGURE 9:**
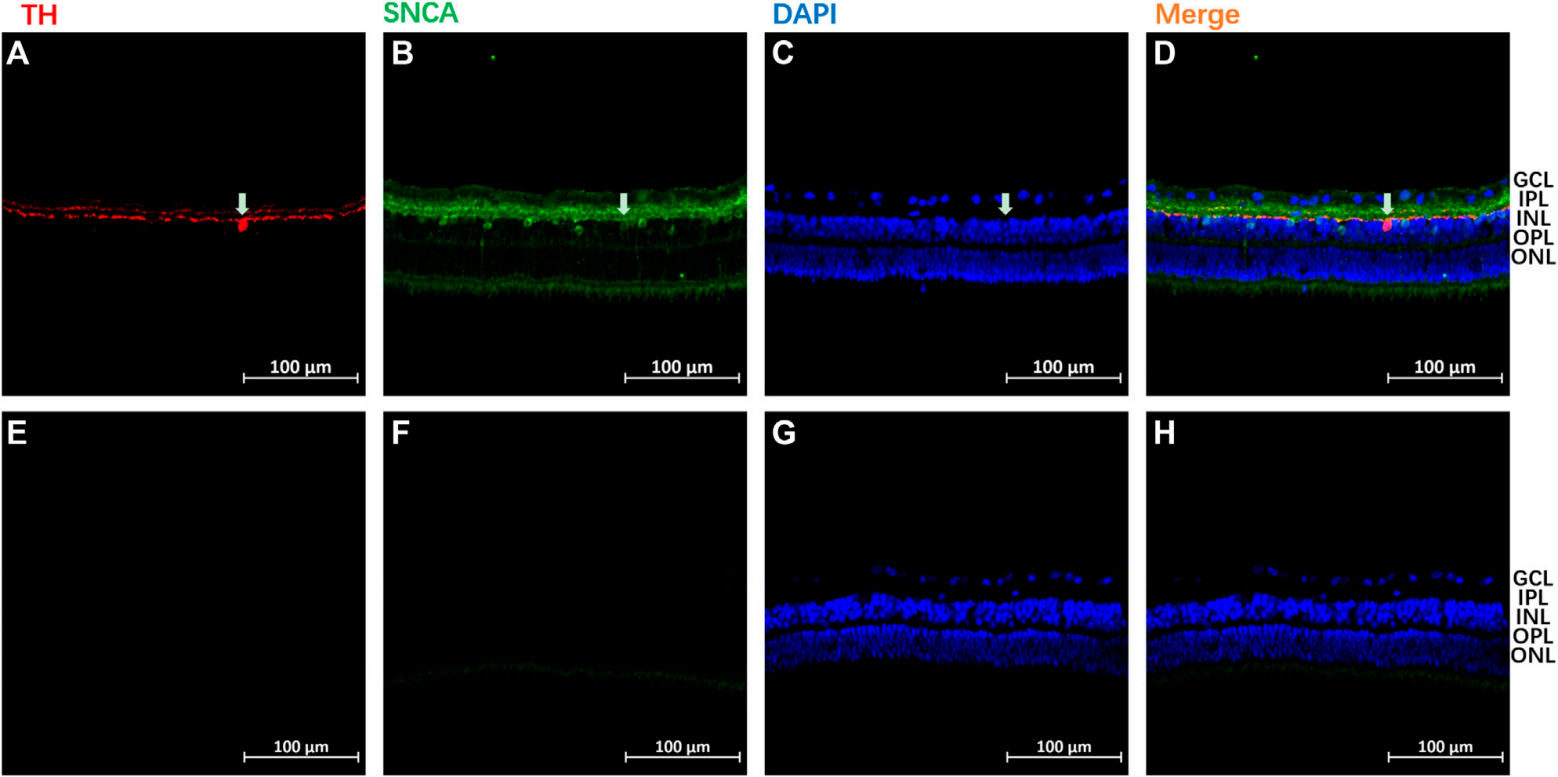
A representative immunoreactivity pattern of α-synuclein (SNCA) and Tyrosine hydroxylase (TH) in the retina of normal guinea pigs. SNCA immune-staining using green, and TH staining using red in panels of **(A–D)**. The panels of **(E–H)** represented the negative controls of staining, slide was not incubated with primary antibodies during staining. The following abbreviations were used: outer nuclear layer (ONL), ganglion cell layer (GCL), inner nuclear layer (INL), outer plexiform layer (OPL), inner plexiform layer (IPL), and outer segments (OS). Each bar equals 100 μm.

The SNCA expression pattern was then analyzed using immunohistochemistry in the retina of normal guinea pigs (n = 3). A similar localization of SNCA was found in all three animals. As shown in Figures 9A–D, high levels of SNCA (stained in green) were found in the inner margin of the inner nuclear layer (INL), and processing spreading throughout the inner plexiform layer (IPL) in the retina. Tyrosine hydroxylase (TH) is a marker for dopaminergic neurons in the central nervous system (Kastner et al., 1993). To further address the intracellular distribution of SNCA and dopamine (DA) in the guinea pig retina, double immunostainings with antibodies to α-synuclein and TH were performed. Colocalization of SNCA (green) and TH (red) was observed in some dopaminergic amacrine cells (white arrows) (Figures 9A–D). The negative control of staining is shown in Figures 9E–H.

## Discussion

### Refractive Error and Ocular Dimensions Changes

After 2-weeks and 4-weeks FDM, relative myopic shifting of refractive errors, elongation of axial length, and vitreous chamber depths observed in the treated eyes were consistent with published parameters of FDM guinea pigs (Lu et al., 2006; Zhi et al., 2021). However, a larger myopic shift (−11.00 ± 0.75 D) in terms of refractive error was found in a previous study in 4-weeks FDM guinea pigs (Wu et al., 2014). This difference in outcomes may be due to the older animals included in the current study. After 2-weeks atropine treatment, less myopia was found in the FDM + A2 group (PN35 to PN49), compared to the FDM group. After a 4-weeks cumulative treatment (PN21 to PN49), notably less myopic shift was observed in the FDM + A4 group than the FDM + A2 group (2-weeks treatment), which indicated a more effective myopia retardation was achieved with a longer period of treatment. For the FDM + A4 group, relative myopic shifting of refractive errors was still observed from PN35 (2-weeks) to PN49 (4-weeks), which implied that longer FDM inducement triggered relatively higher myopia, which was consistent with published parameters from previous FDM studies (Lu et al., 2006). Comparison of the ocular parameters in the FDM + A2 and FDM + A4 groups revealed that the relative hyperopia (less myopia), and a reduced elongation of AL and VCD were found in the FDM + A4 group, which indicated that the myopia control effect of atropine was more effective with extended treatment tie in a doss dependent manner. Studies conducted on children have shown that different concentrations of atropine (low dose, 0.01%; moderate dose, >0.01% to <0.5%; and high dose, 0.5–1.0%) resulted in a slowing down of myopia from 40 to 60% (Gong et al., 2017; Zhao et al., 2020). The current study showed 1% atropine had a slowing down effect on eye growth at PN49 (2-weeks treatment) of 35% in refraction (FDM + A2 *vs* FDM: - 4.55 D *vs.*—6.96 D) and 50% in AL (FDM + A2 *vs* FDM: 0.14 mm *vs* 0.28 mm). A considerably greater myopia control effect of 56% reduction in refraction (FDM + A4 *vs* FDM: -3.05 D *vs.* −6.95 D) and 67% in AL (FDM + A4 *vs* FDM: 0.09 mm *vs* 0.28 mm) was observed at PN49 (4-weeks treatment), which was similar to the findings reported with FDM guinea pigs using 1% atropine through peribulbar injection after 2-weeks treatment (Zhou X et al., 2020) and in children using a moderate dosage of atropine (0.05–0.5%) (Yam et al., 2019; Zhao et al., 2020).

### Protein Identification and Gene Ontology Analysis

For protein identification, an approximately 3-fold more total retinal proteins were observed using SWATH-MS compared to iTRAQ-MS based proteomics. However, it is worth noting that the two approaches resulted in similar GO function classification profiles, especially for the top three functions and processes, representing proteins with high abundance. Using a similar SWATH-MS based MS platform, increases of 46 and 52% in terms of protein IDs and peptide IDs, respectively, were found in the current study, compared to a recent guinea pig retinal study published by our team without use of fractionation techniques (Bian et al., 2021). The results of the current study support the importance of generating a comprehensive spectral library or combining multiple high quality IDA files for a data-independent proteomic approach in biomarker discovery (Palmowski et al., 2019) Comparison with two recent studies of emmetropization (Shan et al., 2018a) and LIM (Bian et al., 2021) in the retina of guinea pigs using the same SWATH approach, revealed very similar GO classification profiles. The GO function classification of retinal proteins, especially the major molecular functions and biological processes observed in our datasets, were consistent with those of previous studies in chicks (Lam et al., 2006), mice (Skeie and Mahajan, 2013), and humans (Velez et al., 2018). The results demonstrate that the overall retinal proteome and GO function classification was consistent and robust across species, regardless of optical or drug treatment.

### Protein Quantitation Using iTRAQ Based Proteomics

The iTRAQ technique is widely employed in proteomic workflows requiring relative quantification for biomarker discovery (Aggarwal et al., 2006). In our iTRAQ based proteomics approach, six retinal lysates from individual animals were randomly pooled under each condition and isobaric labelled for quantitative analysis using a multiplexing approach. A similar pool design was also used in previous proteomics studies in myopic chicks vitreous (Yu et al., 2017) and the LIM mouse retina (Barathi et al., 2014). Using this effective screening design, the individual biological sample variations were reduced to individual protein abundance which was averaged within the same treated group. Also, the pooling approach greatly reduced the experimental running time and costs. In addition, more reliable quantification can be achieved across multiple conditions with reduced materials requirements. More differentially expressed proteins were found in the “FDM + A4 *vs* FDM group” compared to the “FDM + A2 *vs* FDM group”, in line with the hypothesis that more significant changes could occur with a longer treatment period. Despite there being very limited reports of atropine treatment effects on the retina using high throughput omics approaches, the current study using an iTRAQ based proteomic approach found three commonly (Atp1a3, MYEF2, and Hsp90aa1) regulated proteins (FDM + A4 *vs* FDM group) that were also reported in a previous LIM mouse retinal study after a similar 4-weeks atropine treatment (Barathi et al., 2014). Different animal species (mouse *vs* guinea pig), two different paradigms of myopia models (LIM *vs* FDM), and different criteria for detecting differentially regulated proteins may partially account for the discrepancies between the two studies.

For two commonly upregulated proteins, Sodium/potassium-transporting ATPase subunit alpha (*Atp1a3*) is a catalytic component of the active enzyme, catalysing the hydrolysis of ATP coupled with the exchange of sodium and potassium ions across the plasma membrane. A previous study found a high expression of *Atp1a3* in rat and mouse retinas (Wetzel et al., 1999). A recent whole-exome sequencing study reported that high expression of an *ATP1A3* mutation led to cone-rod dystrophy through limiting mitochondrial reserve capacity (Zhou GH et al., 2020). Although abnormal mitochondrial function has been reported during myopia progression, *ATP1A3* had not been previously implicated. Therefore, it may be speculated that up-regulation of *ATP1A3* (FDM + A4 *vs* FDM) may retard myopia by maintaining normal mitochondrial function after atropine treatment. Further research is needed to characterize the relationship between ATP1A3 protein expression and myopia. Increased expression of Myelin Expression Factor 2 (*MYEF2*) was also noted after atropine treatment. *MYEF2* is the transcriptional repressor of the myelin basic protein gene (MBP). Interestingly, in FDM chicks, 38% less myelinated axons were found in the retinal nerve fiber layer after 7-days FDM inducement compared to untreated eyes by means of immunohistochemical labelling against myelin basic protein (Swiatczak et al., 2019). Hence, it can be speculated that the increased expression of *MYEF2* after atropine treatment could inhibit the expression of MBP, leading to a similar result to that seen in FDM chicks. Furthermore, MYEF2 is sensitive to FDM, but not to the atropine treatment. Further studies are needed to determine if MYEF2 is sensitive to LIM. The downregulated protein, Heat Shock Protein 90 Alpha Family Class A Member 1(*Hsp90aa1*) is a subtype of Heat shock protein 90 (HSP90) and acts as a molecular chaperone that catalyses protein folding and maintains “quality control” for a large number of “client” proteins. Although its relationship to myopia is not readily apparent from these functions, a prior study suggested that HSP90 could upregulate the expression of Hypoxia Inducible Factor 1 Subunit Alpha (HIF1α) in senescent ARPE-19 cells and subsequently promote the induction of distinct inflammatory factors (Chen et al., 2021). A previous myopia study also reported upregulation of HIF-1α in the myopic sclera of both mice and guinea pigs (Wu H et al., 2018). Collectively, despite the lack of direct evidence, reports suggest that increased levels of HIFα may contribute to myopia development. The downregulation of *Hsp90aa1* after atropine treatment may reduce HIF1α, thereby slowing down myopia progression.

### Protein Quantitation Using Sequential Window Acquisition of all Theoretical Mass Spectra Based Proteomics

To minimize potential false-positive findings due to the pooling strategy used in the iTRAQ screening approach, a new batch of retinal protein lysates from the FDM and FDM + A4 groups were selected for SWATH-MS based proteomics (*n* = 6) as more significant retarding effects in terms of biometric changes as well as more protein changes were observed with this technique than with the iTRAQ approach. Based on the filtering criteria (FC ≥ 1.4 or ≤0.71, *p*-value of ≤0.05, welch T-test), approximately 770 differentially expressed proteins were detected in FDM + A4 compared to FDM groups. This approach for identifying differentially expressed retinal proteins in the guinea pig FDM model has not been published previously. Only one iTRAQ-based proteomic study using the LIM mouse after 1% atropine treatment (Barathi et al., 2014), and two mRNA expression studies after 100 µM atropine-treated human scleral fibroblasts (Hsiao et al., 2019) and very low dose atropine (0.003%) treatment on corneal epithelial cells (Chang et al., 2019), respectively, have been reported. Specifically, fifteen altered proteins were identified in both studies using the SWATH approach and the previously reported LIM mouse study using iTRAQ-based proteomics. Interestingly, despite the differences in ocular tissues and omics techniques, three (PTPRK, PSMC4, and CIT) commonly regulated proteins were also noted in the transcriptomic study using corneal epithelial cells when compared to those observed in the current study. However, the study of human scleral fibroblast did not show the details of 389 differentially expressed genes with at least a 2.0-fold change. Therefore, no comparison could be performed.

### Pathway and Protein Interaction Analysis of 29 Commonly Regulated Proteins in iTRAQ and Sequential Window Acquisition of all Theoretical Mass Spectra Based Proteomics

By combining iTRAQ-MS and SWATH-MS proteomic approaches, 29 highly confident proteins (at 1% FDR with quantification of at least 2 peptides per protein) were found consistently regulated in response to atropine treatment in the same expression direction. Novel bioinformatics tools were employed for providing molecular insights at systems level. According to the IPA analysis results (Table 2), eukaryotic initiation factor 2 (EIF2) signaling was found to be the most significant pathway, which was supported by clustering by STRING (Figure 6). In previous myopia studies, decreased eukaryotic translation initiation factor 1A was found in the 4-days LIM guinea pig retina (Wu Y et al., 2018). In addition, reduced eukaryotic translation initiation factor 3 subunit 2 beta was identified 7-weeks FDM guinea pig sclera (Zhou et al., 2010). A recent retinal gene-expression study also revealed an association of EIF2 pathway activation with baseline refraction and susceptibility to FDM across eight different strains of mice (Tkatchenko et al., 2019). They reported that negative refractive errors were associated with activation of the EIF2 signaling pathway, and increased susceptibility to FDM was associated with the suppression of EIF2 signaling. Of the three associated down-regulated proteins involved in the EIF2 pathway identified by the current study (Table 2), **60S ribosomal protein L13 (RPL13)** is a large ribonucleoprotein complex responsible for synthesizing proteins in the cell. It has not been previously reported in myopia studies. One study has reported that the DNA methylation changes of RPL13 were associated with the onset of Alzheimer’s disease (AD) (De Jager et al., 2014). In contrast, **40S ribosomal protein S19 (RPS19)** and **40S ribosomal protein S7 (RPS7)** were found up-regulated after atropine treatment in our dataset. They belong to the RPS family, which are components of the ribosome and plays a vital role in controlling translation and cellular homeostasis (Kondrashov et al., 2011). A previous study reported that eIF2A stimulated the initiator methionyl-tRNAi (Met-tRNAMeti) by binding to 40S ribosomal subunits in mouse (Golovko et al., 2016). Therefore, there may be a similar mechanism in guinea pigs. The up-regulation of RPS19 and RPS7 may indicate the activation of the EIF2 pathway after atropine treatment, which then decreases susceptibility to FDM.

Previous studies have confirmed the vital role of another pathway revealed by the STRING analysis (Figure 6): **glycolysis** in the myopic mouse retina (Barathi et al., 2014) and myopic/recovery tree shrew sclera (Frost and Norton, 2012). The roles of three down-regulated proteins observed in atropine control of myopia are also involved in glycolysis. **Pyruvate kinase (PKM)** plays a vital role in glycolysis, as it can catalyze the transfer of a phosphoryl group from phosphoenolpyruvate to ADP, generating ATP and pyruvate. In a cancer study, the increased regulation of PKM2 suggested enhanced glycolysis for tumor cells *in vivo* (Christofk et al., 2008). In addition, an increased level of PKM2 was also reported in chick retina study which compared LIM to LIH (Yu et al., 2020). The current study also found a decreased level of PKM in atropine-treated FDM guinea pigs, confirming the vital role of PKM in both LIM and FDM animal models. Hence, as a cancer study revealed down-regulation PKM, this may indicate that atropine could diminish glycolysis during myopia progression. Another associated protein, **Heterogeneous nuclear ribonucleoprotein D (HNRNPD)** was also found to be significantly decreased after atropine treatment. Heterogeneous nuclear ribonucleoproteins (hnRNPs) represent a large family of RNA-binding proteins. (Zhang et al., 2018). No previous report has implicated HNRNPD in myopia development. A cancer study reported depletion of hnRNPA1 and hnRNPA2 resulted in a concomitant decrease of PKM2 mRNA in Hela and 293 cells (David et al., 2010). Therefore, decreased HNRNPD observed in the current study may also lead to the downregulation of PKM in retinal cells. Retinal glycolysis pathways were recently found to be implicated in the LIM chick myopia model (Yu et al., 2020). Therefore, the effect of atropine on retinal glycolysis should be further explored. Among other isolated candidate proteins, **Basigin (BSG)** is an interesting target worth mentioning. It is a transmembrane protein explicitly expressed by photoreceptors and essential for normal retinal maturation and development. *BSG* mutation has been implicated in early-onset high myopia and predisposed typical myopic phenotypes in human and mutant mice through a trio-based exonic screening study (Jin et al., 2017). Additionally, two previous studies showed that deficiency of this gene led to defective function and photoreceptor degeneration in the retina of mouse (Philp et al., 2003) and mice (Chen et al., 2004). Although the deficiency is different from protein expression changes, BSG was also involved in the binding of rod-derived cone viability factor to the glucose transporter GLUT1, which increases glucose influx into cone photoreceptors. The increased glucose promotes cone survival by stimulation of aerobic glycolysis (Aït-Ali et al., 2015). This evidence implies that the retina may be a target tissue in response to atropine treatment through regulating *BSG* expression. Overall, down-regulation of *PKM, HNRNPD,* and *BSG* may indicate reduced glycolysis in the retina after atropine treatment, which indirectly inhibits accelerated ocular elongation.

Another key molecule found responsive to atropine treatment is Dopamine (DA), which is a neurotransmitter in the retina produced in amacrine cells. DA has long been suggested as a stop signal in myopia research. The retinal DA level was decreased in FDM animal models, including in chicks (Stone et al., 1989), rhesus monkeys (Iuvone et al., 1989), guinea pigs (Mao et al., 2011), and tree shrew (McBrien et al., 2001). A reduced retinal DA level was also found in LIM chicks (Guo et al., 1995). Furthermore, an increasing DA level was reported to prevent FDM in guinea pigs (Mao et al., 2010; Dong et al., 2011), mice (Yan et al., 2015), rabbits (Gao et al., 2006), and monkeys (Iuvone et al., 1991). In addition, a previous study suggested that an intravitreal injection of atropine increased dopamine release and the concentration of its metabolite DOPAC in the chick retina (Schwahn et al., 2000). Recent research also suggested that the metabolism of dopamine was changed by the action of the dysregulated genes after 100 µM atropine-treated human scleral fibroblasts (Hsiao et al., 2019). The findings of these studies confirm the potential association between DA and atropine found in our study. Overall, previous studies had revealed dopamine alteration was involved in myopia development and the common anti-myopic effects of atropine treatment in different species and tissues.

Among the commonly regulated proteins, Alpha-synuclein (SNCA) was identified as the most interesting and novel protein involved in atropine treatment. This protein is involved in multiple pathways and connected with other regulated proteins in the study (Figure 5). SNCA has been reported as a neuronal protein that plays several roles in synaptic activity, such as regulating synaptic vesicle trafficking and subsequent neurotransmitter release. However, SNCA has not been previously implicated in myopia development using conventional molecular approaches. It was considered as a possible biomarker for Parkinson’s disease in human cerebrospinal fluid, plasma, or serum (Hong et al., 2010). It has previously been demonstrated that over-expression of SNCA could directly lead to apoptosis of dopamine neurons and then reduce the striatal DA level in rat primary culture, immortalized mesencephalon-derived cells (Zhou et al., 2000) and transgenic mice (Tofaris et al., 2006). A knockout mice study revealed that SNCA is an essential presynaptic, activity-dependent negative regulator of DA neurotransmission (Abeliovich et al., 2000). Remarkably, the over-expression level of SNCA (up-regulation, 1.92-fold) was comparable to the FDM control group based on iTRAQ-MS based proteomics data. In contrast, a reduced level of SNCA (down-regulation, 0.21-fold, *p* < 0.001) was found after atropine treatment using both approaches. Therefore, our results suggest the involvement of dopamine signaling in the anti-myopic effects of atropine in guinea pig eyes. However, further study is needed to investigate the SNCA effect on inhibiting the apoptosis of dopamine neurons or prompting their secretion.

Based on the immunohistochemistry (IHC) of SNCA and tyrosine hydroxylase (TH), similar patterns of distribution of SNCA in the INL and IPL were reported in the retinas of macaque (Martínez-Navarrete et al., 2007) and humans (Surguchov et al., 2001; Martínez-Navarrete et al., 2007). In addition, the same study (Martínez-Navarrete et al., 2007) also found colocalization of SNCA and glycine in amacrine cells of rat and rabbit retinas. In addition, studies have revealed localization of TH in amacrine cells in the retinas of rabbits (Brecha et al., 1984), rats (Debertin et al., 2015), and humans (Crooks and Kolb, 1992)., DA is released mainly in a paracrine manner by a population of tyrosine hydroxylase expressing (TH+) amacrine cells (AC) of the mammalian retina (Debertin et al., 2015). Taken together, our results allow us to postulate an interaction of SNCA and DA secretion through TH in amacrine cells, which deserves further study.

Of the newly discovered proteins to be differentially expressed in response to atropine treatment, a decrease (down-regulation, 0.82-FC) of Mitochondrial import receptor subunit TOM22 homolog (TOMM22) was observed in the myopic retina, and the increased expression change (up-regulation, 1.4-FC, *p* < 0.05) was found after atropine treatment. *TOMM2* was associated with mitochondrial dysfunction. It is a central receptor component of the translocase of the outer membrane of mitochondria (TOM complex) and is responsible for the recognition and translocation of cytosolically synthesized mitochondrial preproteins. *TOMM22* has not been previously implicated in myopia development. However, a recent paper showed that yeast mitochondrial accumulation of amyloid β (Aβ) peptides requires *TOMM22* as the main Aβ receptor (Hu et al., 2018). Although abnormal mitochondrial function has been reported during myopia progression, amyloid β (Aβ) peptides and TOMM22 have not been previously implicated (Wojciechowski, 2011). One recent RNA seq study also reported increased mitochondrial metabolism was found in chicks 24 h after 7-days FDM chicks, which explained why dark-adapted (low temporal luminance modulation) retinae require ∼ 20% more metabolic activity than the same light-adapted retina (Vocale et al., 2021). Therefore, our finding also highlighted the importance of characterizing the relationship between mitochondrial cascades and the anti-myopic effects of atropine.

The main limitation of this study was the use of sample pooling for the iTRAQ-MS proteomic approach, potentially increasing the risk for false-positive findings (e.g., by outlier effects). However, this risk was mitigated via SWTAH-MS proteomic analysis with individual biological samples (*n* = 6), which required differentially expressed proteins to be identified in consistent directional regulation of both proteomic analyses with significance in SWATH-MS proteomic analysis. The study also yielded technical comparisons of iTRAQ-MS and SWATH-MS based proteomics. Eight samples can be analyzed in one experiment using iTRAQ (Ross et al., 2004). After labeling, eight labels were combined and injected into MS together and analyzed simultaneously in DDA, which reduced the run-to-run variation and analysis time. Similar behavior of peptides, including retention time, the efficiency of ion, and response to electron spray ionization (ESI), has been observed (Xie et al., 2011). However, the limitations of DDA are bias of under-sampling low abundance precursor ions (Stahl et al., 1996), the potential difference in labeling efficiencies, and digestion effects (Elliott et al., 2009), which may contribute to fewer proteins being identified using iTRAQ. As SWATH-MS is a specific variant of the DIA method (Ludwig et al., 2018). All the peptides are fragmented in MS/MS, regardless of peptide intensity (Chapman et al., 2014). As a result, compared with DDA, data reproducibility between technical replicates is increased, and low abundance precursors are better represented (Geromanos et al., 2009). Hence, the total number of proteins identified was three times higher using compared iTRAQ-MS based proteomics.

For SWATH-MS proteomics, fewer procedures are required without the need for the labeling of peptides. In addition, there is no limitation of the total sample number. Once the peptides are prepared, they are injected into LC-MS for analysis, and each sample runs individually. Therefore, a large number of biological samples can be analyzed by this more straightforward approach (Wasinger et al., 2013).

One of the most interesting, regulated proteins, the localization of SNCA was confirmed in the current study. Further validation experiments are needed to confirm the protein changes of SNCA, PKM, and BSG related to the atropine treatment in FDM guinea pigs. Based on our integrated label and label-free proteomics analysis, more targeted and specific pathways were identified for the first time in the FDM guinea pig retina in response to atropine treatment. Our established protocols confirmed the feasibility of applying high throughput proteomics for investigating novel mechanisms in the therapeutic treatment of myopia. Similar studies should be performed in the FDM or LIM guinea pig retina using a low dosage of atropine (0.01,0.05, and 0.1%) in the future to determine if common regulatory mechanisms could be repeated in different myopia paradigms and different dosages being used in human myopia control (Chia et al., 2012; Yam et al., 2019). In addition to analyzing retinal tissue, this combined iTRAQ-MS and SWTAH-MS proteomics approach can also be used to investigate potential protein signals in other ocular structures from the vitreous to the sclera for much more complete identification and quantitation of biomarkers and pathways at the posterior eyes in response to atropine treatment during myopia progression.

## Materials and Methods

### Animals

Pigmented Guinea pigs (*Cavia porcellus,* the English short-hair stock, Danyang Changyi Experimental Animal Center Co., Ltd., China) were raised with their mother till postnatal day 18 (PN 18). All treatment and care of animals complied with the ARVO Statement, and the protocol for handling animals was in accordance with NIH Guidelines. The experimental period started from postnatal 3 weeks (PN 21) to 7 weeks (PN 49). The FDM model was established based on a published protocol (Lu et al., 2006). Guinea pigs were raised in standard cages (65 × 45 × 20 cm) at 25°C with sufficient food, water, and fresh vegetables daily. With lights on 8:00 AM, the 12 h:12 h light/dark cycle was controlled by straight fluorescent lamps, and the central ambient luminance over the cages was maintained around 300 lux. Animals were randomly assigned to four groups, including normal control group (NC, *n* = 7), monocular form-deprivation myopia group (FDM, *n* = 7), FDM with 2-weeks atropine-treatment group (FDM + A2, *n* = 7), and FDM with 4-weeks atropine-treatment group (FDM + A4, *n* = 8). For the NC animals, both eyes were exposed naturally without any lens attachment; For the FDM group, either the right or left eye was randomly covered by a white latex facemask, leaving the contralateral eye, nose, mouth, and ears freely exposed from PN 21 to PN 49 (4 weeks FDM). In the atropine treatment groups, 10 g L^−1^ atropine gel (Xingqi Pharmaceutical Co. Ltd., China) was topically administered to the FDM eyes from PN35 to PN49 (FDM + A2 group, 2 weeks drug treatment) and from PN21 to PN49 (FDM + A4 group, 4 weeks drug treatment), respectively. Body weight and ocular biometrics were recorded at PN21, PN35, and PN49. For iTRAQ-MS based proteomics study, retinas from all twenty-nine guinea pigs were collected at PN49 (Figure 8). For SWATH-MS based proteomics study, retinas of another cohort of twelve guinea pigs (FDM group: *n* = 6, FDM + A4 group: *n* = 6) were also harvested at PN49 after 4 weeks treatment.

### Ocular Biometric Measurements

The steak retinoscopy (66 vision. Tech, China) and A-scan ultrasonography (KN 1800, Kangning Medical electronic equipment development company, China) were used to measure refractive error and ocular dimensions, respectively. 0.5% Compound Tropicamide (Xingqi Pharmaceutical Co. Ltd., China) was used to paralyze the ciliary muscle and dilate the pupil. The refractive error was recorded as the average of 3 repeated measurements of spherical equivalent (SE), which is calibrated by the sum of spherical value and half of the cylindrical value. 0.4% Qxybuprocaine Hydrochloride (Santen Pharmaceutical Co. Ltd., Japan) was used for corneal anesthesia to reduce the discomfort of animals from the ultrasonic probe. The axial length (AL), anterior chamber depth (ACD), lens thickness (LT), vitreous chamber depth (VCD) was obtained by A-scan, which were averaged from 10 repeated measurements. All the measurements were performed by the same optometrist. The facemask was re-attached immediately after measurements.

### Retinal Harvest and Protein Extraction

Guinea pigs were sacrificed at PN49 by cervical dislocation. The anterior segment, crystalline lens, and vitreous from the eye cup were removed, the retina was carefully peeled off from the posterior hemisphere without retinal pigment epithelium within 5 min as previously reported (Anjo et al., 2017). Two kinds of lysis buffer were included in this study to extract protein from retinal tissue. For the iTRAQ approach, 200 µl of Nitroextra (9 M Urea, SDS, Tritone X, and Protease inhibitors) was added into each frozen tissue. Samples were pooled and blended with sonication for 5 min. For the SWATH approach, collected retinal tissues were homogenized using liquid nitrogen-cooled Precellys^®^ Evolution homogenizer (Bertin Technologies) with 200 µl of customized SDS lysis buffer (5%SDS, 50 mM TEAB, pH7.55) at 5,800 rpm for 120 s at 4°C. The recovered retinal lysates were then centrifuged at 21,380 × g for 30 min at 4°C, followed by collecting and storing the supernatant in a new tube at −80°C until further analysis. The total protein concentration was measured by Pierce™ Rapid Gold BCA protein assay (Thermo Fisher Scientific, United States) according to the manufacturer’s protocol.

### Protein Digestion and iTRAQ Labeling

Protein was cleaned up by pre-cooled acetone (320110, Sigma) and incubating at −20°C overnight. Then the protein pellet was washed with pre-cooled acetone and dried in a biosafety cabinet. Proteins were resuspended in 8 M urea and reduced with 20 mM dithiothreitol (DTT) at 60°C for 1 h, then alkylated with 40 mM iodoacetamide at room temperature for 30 min protected from light. Samples were diluted to 2 M urea and digested with trypsin in a 1:100 (w: w) ratio at 37°C overnight. Approximately 100 µg aliquots of pooled desalted peptides from each experimental condition were chemically labeled with iTRAQ 8-plex reagent (4466096, SCIEX) in 100 mM TEAB. Labels were arranged as follows: 113: right eyes of NC; 114: left eyes of NC; 115: fellow control eyes of FDM + A2; 116: treated eyes of FDM + A2; 117: fellow control eyes of FDM + A4; 118: treated eyes of FDM + A4, 119: fellow control eyes of FDM; 121: treated eyes of FDM (Left panel in Figure 3). After the labeling reaction at room temperature for 2 h, all the labeled samples were cleaned up by C18 desalting. Differentially labeled peptide samples were resuspended in buffer A (10 mM KH2PO3, 20% acetonitrile (ACN), pH2.7). SCX chromatography was performed with a Poly SULFOETHY A™ (200 × 4.6 mm, 200 A) column using step gradients (0–10 min: 0%B, 29 min: 15%B, 44 min: 45%B, 46–53 min: 100%B) of Buffer A and B (10 mM KH2PO3, 20%ACN, 0.6 M KCl, pH2.7) and a flow rate of 1 min/ml. Fifty-three fractions in total were collected. Samples were further combined into five fractions based on the number of proteins identified in each 10 min fraction. Each fraction was desalted with ZipTip (Cat. ZTC18S960, Millipore) and dried in a spin vacuum for further LC-MS/MS analysis.

### LC-MS/MS Analysis for iTRAQ

The dried peptide was dissolved in 0.1% formic acid (FA). About 3 µg peptides were analyzed by Eksigent ekspert™ nanoLC 425 system coupled to a TripleTOF 6,600 System (SCIEX, MA, United States). The peptides were trapped (ChromXP nanoLC Trap column 350 μm × 0.5 mm, ChromXP C18 3 μm) and eluted at a flow rate of 300 nL/min into a reverse phase C18 column using a linear gradient of ACN (3–36%) in 0.1% FA with a total run time of 120 min. The tandem mass spectra were recorded in positive-ion and “high-sensitivity” mode with a resolution of ∼35,000 full-width half-maximum. Advanced DDA was used for MS/MS collection on the Triple TOF 6600 to obtain MS/MS spectra for the 20 most abundant and multiply charged (z = 2, 3, or 4) following each survey MS1 scan, allowing typically for 250 msec acquisition time per each MS/MS. After 2 repetitive occurrences, the dynamic exclusion was set for 30 s.

### Protein Digestion in Sequential Window Acquisition of all Theoretical Mass Spectra Analysis

Proteins were digested as described recently for the mouse retina (Sze et al., 2021). In brief, a total of 50 μg proteins were reduced with DTT for 10 min, then alkylated with IAA at room temperature for 10 min with protection from light. The alkylation reaction was quenched by adding 0.2% phosphoric acid. Samples were added to the S-Trap protein binding buffer, and proteins were trapped by a filter in the S-Trap Micro Spin Column (Protifi, United States) (HaileMariam et al., 2018). After trypsin (Promega, United States) digestion, the peptides were resuspended with 0.1% FA for LC-MS/MS analysis (calibrated at 0.5 μg/μl) using Pierce Quantitative Colorimetric Peptide Assay (Thermo Fisher Scientific, United States).

Both DDA and SWATH-MS analyses were performed by the TripleTOF 6,600 system (SCIEX, MA, United States) connected to an Eksigent ekspert™ nanoLC415 system similar to our previous protocols (Shan et al., 2018b; Cheung et al., 2020; Bian et al., 2021). For either IDA or SWATH acquisitions, 2 µg peptide was loaded to a trap column (100 μm × 2 cm, C18) for 15 min. Then, it was separated on a nano-LC column (100 μm × 30 cm, C18, 5 µm). An isolation of 100 Variable windows was selected in a looped mode over the full mass range of 100–1800 m/z scan in SWATH acquisition.

### Ion Library Generation for Sequential Window Acquisition of all Theoretical Analysis

Peptides from 12 guinea pigs (FDM and FDM + A4 groups, *n* = 6 per group) were pooled together and then divided into six fractions using Pierce™ High pH Reversed-Phase peptide fractionation kit (84868, Thermo Scientific, United States). Six separate IDA injections were combined to generate an ion library (.group file) for SWATH analysis. It was searched against the guinea pig Uniprot database in ProteinPilot™ (v5.0, SCIEX, MA, United States) software utilizing the Paragon algorithms with the following parameters, identification as sample type, iodoacetamide as Cys alkylation, trypsin digestion, thorough search effort, and with FDR analysis activated. The resulting group file was used as the ion library file for all SWATH files processing and quantification.

### Sequential Window Acquisition of all Theoretical Mass Spectra Acquisitions and Processing

Two micrograms (2 µg) of all 24 biological samples (both eyes of FDM group and FDM + A4 group) with two technical replicates each were injected for SWATH-MS quantification. The generated raw data (.wiff) were processed with PeakView (V2.2, SCIEX, MA, United States) to extract relevant transitions of each identified peptide/protein using the generated combined ion library. Fifteen peptides with high signal/noise ratios were selected for retention time calibration. The resulting data were exported to MarkerView (V1.3.1, SCIEX, MA, United States) for normalization using the MLR method (Lambert et al., 2013), followed by statistical analysis.

### Bioinformatics Analysis and Pathway Analysis for Differentially Expressed Proteins

The Universal Protein Resource online database (UniProt, http://www.uniprot.org/, 9-March-2020) was used to convert protein names to gene names. Functional analysis of Gene Ontology (GO) annotations of identified retinal proteins was performed using the PANTHER gene classification analysis software (PANTHER™ version 15.0, http://pantherdb.org/, 9-March-2020) (Mi et al., 2019). The STRING v11.0 (https://string-db.org/,13- July -2021) database was used to analyze the protein interaction of commonly regulated proteins by two approaches (Szklarczyk et al., 2019). The Ingenuity Pathway Analysis (IPA, Ingenuity Systems, Mountain View, CA, United States, 6—July—2021) was used for pathway analysis (Guerra, 2008).

### Immunohistochemistry and Confocal Imaging

Guinea pigs were sacrificed by cervical dissection. After removing muscle and anterior segments of the eye, eyecup was fixed using 4% paraformaldehyde (P0099, Beyotime, China) for 0.5 h, followed by impregnating using 10% sucrose in PBS for 2 h, 20% sucrose for 2 h, and then 30% sucrose for 15 h, and finally embedding into Optimal Cutting Temperature (OCT) compound to freeze immediately with liquid nitrogen. The embedded tissue was sectioned into 10 μm thickness for immunofluorescence analysis. Retina slides were blocked with QuickBlock™ solution (P0260, Beyotime, China) for 1 h, then the primary antibodies (Mice anti-SNCA, 1:500, AHB0261, Invitrogen, United States; Rabbit anti TH, 1:1,000, AB152, Sigma-Aldrich, United States) were diluted with a blocking solution (P0262, Beyotime, China) and used to incubate retinas for 16 h at 4°C. After incubating and rinsing, secondary antibodies conjugated to Goat anti-Rabbit-Cy3 (1:1,000, A0516, Beyotime, China) and Goat anti-Mice-Alexa Fluor 488 (1:1,000, ab150113, Abcam, United States) were applied for 2 h at room temperature. Zeiss LSM800 (Carl Zeiss, Germany) and a confocal microscope was used to take micrographs at 10 × 20 fold.

### Statistical Analysis

The biometric parameters in the normal control group, FDM group, FDM + A2 group, and FDM + A4 group were analyzed using Two-way mixed design ANOVA with Bonferroni multiple comparisons as previously reported (Kang et al., 2018) using the R program (v4.0.5, Shake and Throw) (Mair et al., 2015). The inter-group differences were defined as significant at *p* < 0.05 and highly significant at *p* < 0.01. ProteinPilot™ (v5.0, SCIEX, MA, United States) was used for iTRAQ analysis. The error tolerance for precursor mass was 15.0 ppm and fragment ion 0.2 Da. To identify the proteins with the most robust differential expression using iTRAQ based proteomics, the criteria of differentially expressed proteins had to fulfill the following criteria: 1) Proteins had an expression fold change (FDM + A2 *vs* FDM or FDM + A4 vs FDM) ≥1.5 or ≤0.67; 2) Proteins had a 1% FDR with at least 1 peptide with 95% confidence identified. Considering the high repeatability of SWATH-MS based proteomics (Collins et al., 2017), filtering criteria in SWATH-MS [proteins with fold change (FDM + A4 *vs* FDM) ≥1.4 or ≤0.71, *p*-value of ≤0.05, welch T-test) to determine significant regulation was lower than the cut-off threshold of the iTRAQ-MS approach, the cut-off threshold was the same as described in our previous published paper of guinea pig retina using the same approach (Shan et al., 2018a).

## Conclusions

In summary, the guinea pig model for FDM was successfully established with documentation of biometric parameter changes under our experimental conditions. In addition, the inhibitory effect of 1% atropine on FDM progression was also observed at two treatment time points. This study built the first FDM guinea pig retina proteome covering myopia development and atropine treatment with the largest high quality retinal proteome with 5,961 proteins (51,871 peptides) reported to date. Using iTRAQ-MS based proteomics for multiplex screening, hundreds of differential protein expressions were identified and quantified. Combining our established SWATH-MS protocol for orthogonal validation, 29 commonly regulated proteins were highlighted as promising targets in response to effective atropine treatment for the first time. In addition, the potential biological pathways involved in the anti-myopic effects of atropine were screened through index proteins, including EIF2 signaling, glycolysis, and regulation of dopamine. The localization of a key retinal protein (SNCA), which could play an important role involve in anti-myopic treatment of atropine, was further confirmed. In addition, other differentially expressed proteins explored in this study may have pivotal roles in the development of FDM and may be responsive to the atropine myopia control treatment. However, whether low dose Atropine (0.1%) treatment shares similar molecular pathways warrants further investigation. In conclusion, the present work has demonstrated the feasibility of using a combined iTRAQ-MS and SWATH-MS proteomic approach for exploring ocular drug treatment effects in a high-throughput manner.

## Data Avilaibility Statement

The raw data presented in this study are available on PeptideAtlas public repository (Zhao et al., 2020) with the accession number of PASS01507 for open access (http://www.peptideatlas.org/).
