# Supplementary material for: Alteration of EIF2 Signaling, Glycolysis, and Dopamine Secretion in Form-Deprived Myopia in Response to 1% Atropine Treatment: Evidence From Interactive iTRAQ-MS and SWATH-MS Proteomics Using a Guinea Pig Model
Source: Front Pharmacol. 2022 Jan 28;13:814814. doi: 10.3389/fphar.2022.814814 (PMC8832150; doi:10.3389/fphar.2022.814814)
Supplement: Supplementary file 1 [file DataSheet1.PDF]

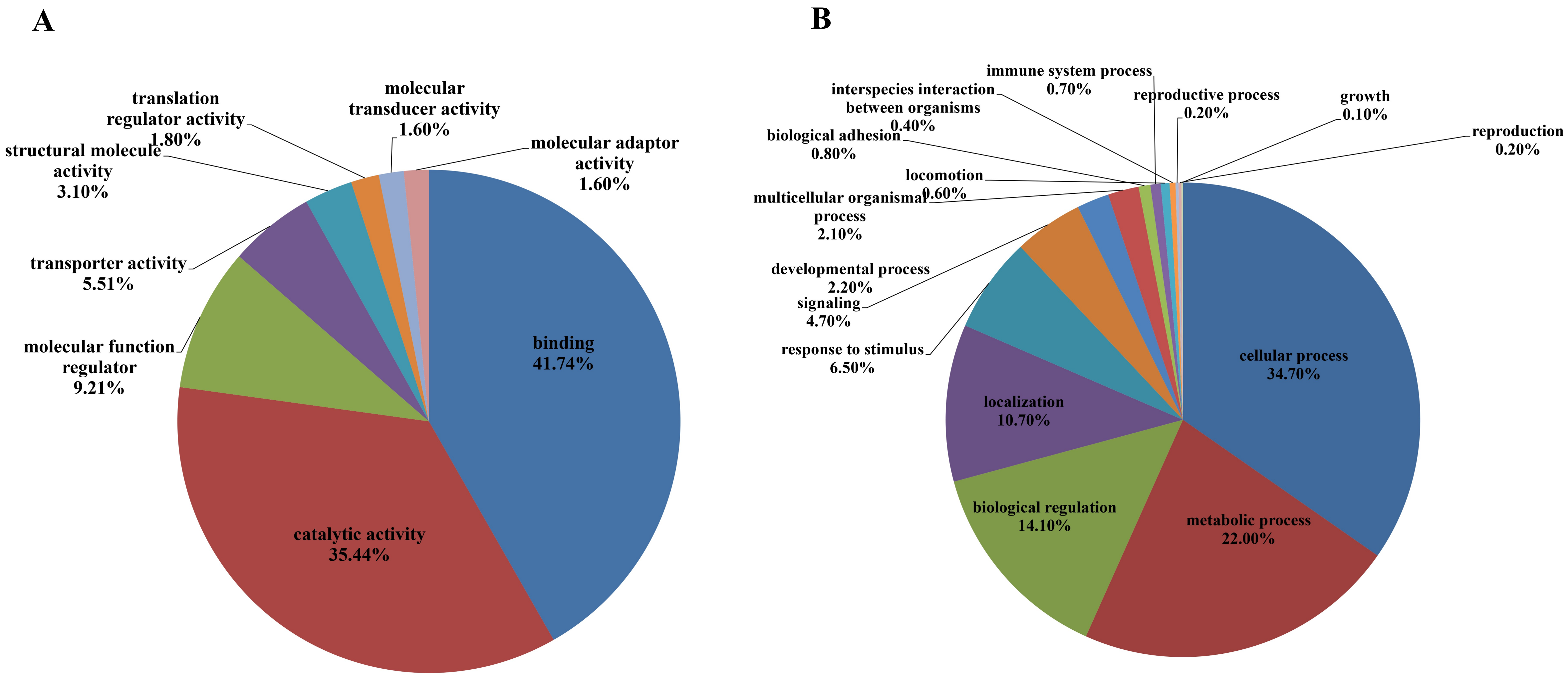

Supplementary Figure 1. A total of 774 differentially expressed proteins (714 gene names) after SWATH-MS quantitative analysis were annotated using the PANTHER^{TM} Classification System, according to their molecular function **(A)** and biological process **(B)**.
